# A Monoclonal Antibody to *O*-Acetyl-GD2 Ganglioside and Not to GD2 Shows Potent Anti-Tumor Activity without Peripheral Nervous System Cross-Reactivity

**DOI:** 10.1371/journal.pone.0025220

**Published:** 2011-09-22

**Authors:** Nidia Alvarez-Rueda, Ariane Desselle, Denis Cochonneau, Tanguy Chaumette, Béatrice Clemenceau, Stéphanie Leprieur, Gwenola Bougras, Stéphane Supiot, Jean-Marie Mussini, Jacques Barbet, Julie Saba, François Paris, Jacques Aubry, Stéphane Birklé

**Affiliations:** 1 Centre de Recherche en Cancérologie de Nantes Angers, Inserm, Université de Nantes, Nantes Atlantique Universités, U892, France; 2 UFR des Sciences Pharmaceutiques et Biologiques, Université de Nantes, Nantes Atlantique Universités, France; 3 Centre de Référence des Maladies Neuro-Musculaires Rares Nantes-Angers, Centre Hospitalier Universitaire de Nantes, France; 4 Children's Hospital Oakland Research Institute, Oakland, California, United States of America; Institute of Cancer Research: Royal Cancer Hospital, United Kingdom

## Abstract

**Background:**

Monoclonal antibodies (mAb) against GD2 ganglioside have been shown to be effective for the treatment of neuroblastoma. Beneficial actions are, however, associated with generalized pain due to the binding of anti- GD2 mAbs to peripheral nerve fibers followed by complement activation. Neuroblastoma cells that express GD2 also express its *O*-acetyl derivative, *O*-acetyl- GD2 ganglioside (*O*AcGD2). Hence, we investigated the distribution of *O*AcGD2 in human tissues using mAb 8B6 to study the cross-reactivity of mAb 8B6 with human tissues.

**Methodology/Principal Findings:**

The distribution of *O*AcGD2 was performed in normal and malignant tissues using an immunoperoxydase technique. Anti-tumor properties of mAb 8B6 were studied in vitro and in vivo in a transplanted tumor model in mice. We found that *O*AcGD2 is not expressed by peripheral nerve fibers. Furthermore, we demonstrated that mAb 8B6 was very effective in the in vitro and in vivo suppression of the growth of tumor cells. Importantly, mAb 8B6 anti-tumor efficacy was comparable to that of mAb 14G2a specific to GD2.

**Conclusion/Significance:**

Development of therapeutic antibodies specific to *O*AcGD2 may offer treatment options with reduced adverse side effects, thereby allowing dose escalation of antibodies.

## Introduction

Neuroblastoma is a cancer of the sympathetic nervous system that is responsible for 12% of deaths associated with cancer in children under 15 years of age [1]. Despite advances in the treatment of low- to intermediate-risk neuroblastoma, outcomes for patients diagnosed with a high-risk phenotype characterized by widespread dissemination of the cancer remains poor. Patients undergo relapse and ultimately die from the tumor in spite of the standard aggressive treatment, which includes surgery, radiation, and/or myeloablative chemotherapy with stem cell rescue, followed by 13-*cis*-retinoic acid [2]. Thus, once remission is achieved, the major obstacle to a cure is residual chemotherapy-refractory disease that eludes current methods of detection [3].

One of the most promising approaches for increasing the efficiency of standard therapy in this case involves anti-ganglioside GD2 immunotherapy [4]. GD2 ganglioside is an acidic glycosphingolipid that is abundantly expressed on the cell surface of tumor cells of neuroectodermic origin such as neuroblastoma [5]. In normal tissue, GD2 expression is largely limited to neurons, skin melanocytes and peripheral nerve fibers [6], [7], making it well suited for targeted antitumor therapy. The rationale for passive immunotherapy with anti-GD2 mAbs is supported by their anti-tumor properties. Preclinical studies have shown that anti-GD2 mAbs may inhibit tumor cell growth via direct cell death induction [8]. In addition, anti-GD2 mAbs can mediate tumor cell destruction through antibody-dependent cellular cytotoxicity (ADCC) and complement cell cytotoxicity (CDC) [9], [10]. Interestingly, GD2 has recently been ranked 12^th^ in priority of all clinical antigens by an NCI workshop [11].

Several anti-GD2 antibodies have been developed for clinical use over the past 2 decades, two of which are under evaluation in the clinical setting: ch14.18 [4] and 3F8 [12]. 3F8 is a completely murine antibody and ch14.18 is a human–mouse chimeric construct consisting of variable regions derived from the murine anti-GD2 antibody 14G2a and of constant regions of heavy and light chains from a human IgG1 molecule. A recent phase III trial has shown that a combination of anti-GD2 ch14.18 antibody and cytokines with the standard therapy significantly improved outcome [4]. Although these results are very encouraging, one of the major drawbacks of anti-GD2 mAbs is their toxicity. The infusion is frequently associated with severe pain, changes in cardiovascular tone, fever and complement depletion [13], [14]. Furthermore subsequent to treatment with anti-GD2 monoclonal antibody some patients have developed sensorimotor polyneuropathy [15]. These neurotoxic toxicities are most likely the result of mAb recognition of GD2 on pituitary gland and peripheral nerves and complement activation [6]. Hence, they limit the dose of anti-GD2 mAbs that can be given and therefore its clinical efficacy.

In an effort to increase the therapeutic index of ch14.18, a humanized antibody was recently designed in which the Fc region was mutated in the CH2 domain to no longer engage C1q. The resultant antibody, hu14.18 K322A, retained potent ADCC activity against GD2-expressing tumor with impaired complement activation in vitro, and, reduced neurotoxic side effects in rat [16]. However, since it still retains its binding activity to peripheral nerve fibers, this format would not be suitable for developing immunotherapeutic agents by conjugation to toxins, radionuclides or other effector molecules.

In our laboratory, several anti-disialo-gangliosides antibodies have been generated that recognize GD2, GD3 and acetylated GD2 and GD3. One of these antibodies, mAb 8B6, was shown to be specific for the *O*-acetylated derivative of GD2 (*O*AcGD2) with no cross-reaction with GD2 [17] by thin layer chromatography (TLC) immunostaining. *O*AcGD2 is concomitantly expressed by GD2-positive tumor cells [18], [19]. This antibody, mAb 8B6, was not found to cross react with GD3, acetylated GD3 or other gangliosides [17]. This prompted us to investigate the distribution of OAcGD2 in human tissues. In contrast to GD2, *O*AcGD2 is not expressed by human peripheral nerve fibers, suggesting that anti-OAcGD2 8B6 antibody has the potential to be less toxic than anti-GD2 therapeutic antibodies. In addition, mAb 8B6 is able to inhibit tumor cells growth in vitro and in vivo. Interestingly, the anti-tumor activity is comparable to anti-GD2 mAb 14G2a. These results highlight the use of antibodies that target specifically *O*AcGD2 to avoid the side effects of mAbs to GD2 in patients.

## Results

### Reactivity of mAb 8B6 with peripheral nerves and other different normal tissues

#### Peripheral Nerves

**Figure 1:**
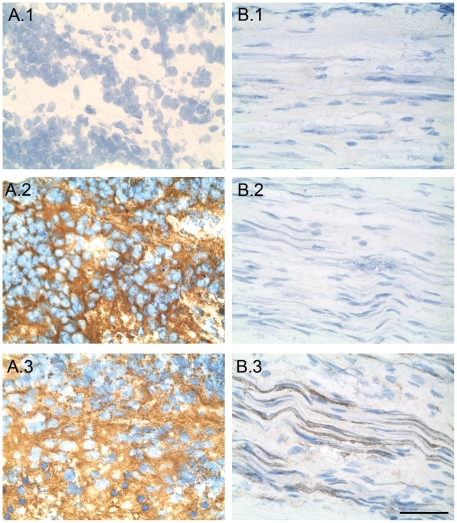
Expression of *O*AcGD2 and GD2 ganglioside antigens on peripheral nerves and neuroblastomas. An immunoperoxydase assay was performed as described in Material and Methods. Strong immunostaining was detected on neuroblastoma cells (A) with either mAb 8B6 (2) or mAb 14G2a (3). No staining was observed on peripheral nerves (B) with mAb 8B6 specific for *O*AcGD2 (2). Myelin sheaths in the peripheral nerves (B) were strongly stained with mAb 14G2a against GD2 (3). The mouse IgG3 mAb negative control from AbD serotec (Oxford, UK) was used as a negative control (1). Scale bar  = 50 µm.

Binding of mAbs specific for GD2 to peripheral nerves with subsequent activation of the complement cascade is suspected to induce toxic effects when they are administrated in patients. This prompted us to study the reactivity of anti-*O*AcGD2 mAb 8B6 against peripheral nerves with an immunoperoxydase technique. Example of results obtained on peripheral nerves and neuroblastomas are shown in Fig. 1 B.2. When anti-*O*AcGD2 mAb 8B6 was tested on all of the 12 different samples, no labeling was present. Axons were negative. By contrast, the sections of all the 12 different samples stained with anti-GD2 mAb 14G2a showed strong positive staining of the nerve fibers as shown in Fig. 1-B.3. The mouse isotype control antibody was also negative (Fig. 1 B.1). These data show that mAb 8B6 do not bind to peripheral nerves and suggest that antibodies specific to *O*AcGD2 may offer new treatment options with reduced adverse side-effect compared to anti-GD2 mAbs. Five other representative results are depicted in Fig. S1.

#### Other Normal Tissues

We next evaluated the reactivity with other human tissues. Table 1 summarizes the reaction pattern obtained when mAb 8B6 was tested against the 32 normal tissues recommended by the FDA. Peripheral blood erythrocytes and leukocytes, bladder, breast, brain cortex, fallopian tube, eye, heart, liver, pituitary (Fig. S2), ovary, pancreas, prostatic epithelium, skin, spleen, testis, thymus, ureter, vascular endothelium and smooth muscle, nerves, and uterine cervix, endometrium, and myometrium tissues were all negative. In the adrenal, the zona glomerulosa and fasciculata were negative while the zona reticularis showed faint cytoplasmic staining and moderate granular intracellular staining (Fig. S2). There was also slight reactivity with Purkinje cells and the Bergmann glia in the cerebellum (Fig. S2). Antibody 8B6 reacted with lymph node germinal center cells (Fig. S2). In the bone marrow, antibody 8B6 did not show any binding to the erythroid, myeloid, and megakaryocyte series. Occasional macrophages showed moderate granular cytoplasmic staining (Fig. S2). Antibody 8B6 also reacted faintly with the dorsal horns in the spinal cord, and subsets of thyroid follicular epithelial cells (Fig. S2). These data indicated that mAb 8B6 presents a very interesting safety reactivity profile for its clinical use.

**Table 1:** *O*AcGD2 expression on normal tissues defined by immunohistology*.

Tissue	mAb 8B6 binding to frozen tissue sections
Adrenal	Negative†
Bladder	Negative
Blood Cells	Negative
Bone Marrow	Negative^I^
Brain, Cerebellum	Negative‡
Brain, Cortex	Negative
Breast	Negative
Colon	Negative
Endothelium	Negative
Eye	Negative
Fallopian Tube	Negative
Gastroinstestinal Tract	Negative§
Heart	Negative
Kidney, Cortex and Medulla	Negative
Liver	Negative
Lung	Negative
Lymph Node	Negative^II^
Ovary	Negative
Pancreas	Negative
Parathyroid	Negative
Pituitary	Negative
Placenta	Negative
Prostate	Negative
Skeletal Muscle	Negative
Skin	Negative
Spinal Cord	Negative**
Spleen	Negative
Testis	Negative
Thymus	Negative
Thyroid	Negative††
Ureter	Negative
Uteris, Cervix	Negative
Uterus, endometrium	

*Determined by immunoperoxydase assay using mAb 8B6 as described in Material and Methods. The number of each tissue section tested ranged from 2 to 3.

† The zona reticularis showed faint cytoplasmic staining and moderate granular intracellular staining;

I Occasional macrophages were positive

‡ the Purkinje Neurons were faintly positive;

§ the epithelium apical surface showed staining similar to that of the isotype control antibody;

II; the germinal centers of lymphoid follicles showed moderate to strong stainning;

**the gray matter in the dorsal horns showed moderate staining;

†† the follicular epithelium showed faint cytoplasmic staining.

#### Tumor tissues

*O*AcGD2 was originally isolated from neuroectodermic tumors such as neuroblastoma and melanoma [18], [19]. We, therefore, studied the binding activity of mAb 8B6 to several human malignant tissues (Table 2). Table 2 summaries mAb 8B6 immunoreactivity on tumor samples. As mentioned above, an example of neuroblastoma tumor section stained by mAb 8B6 is shown in Fig. 1. Other examples of neuroblastomas are depicted in Fig. S3 and other *O*AcGD2-expressing tumors are shown in Fig. S4. By IHC analysis, mAb 8B6 bound to 12/12 neuroblastomas, 3/4 small cell lung carcinoma and 3/4 melanomas. In positive tumors, 100% of tumor cells in neuroblastomas were stained (Fig. S3), 75% for small cell lung carcinomas, and 50% for melanomas. Staining was strongly membranous and also faintly cytoplasmic (Fig. S4). Tumor tissues from patients with pancreatic carcinomas (n = 5) were negative. Ganglioside *O*AcGD2 expression was also evidenced in the human IMR32 neuroblastoma passaged in nude mice, the mouse EL4 T-lymphoma grafted in C57Bl/6 mice, and the mouse NXS2 neuroblastoma grafted in A/J mice (data not shown).

**Table 2:** Presence of *O*AcGD2 in human tumors determined by immunoperoxydase assays*.

Tumor tissue	# of positive tumor tissues / # of tumor tissues tested	% of positive cells in OAcGD2-positive tumors
Neuroblastoma	12/12	100%
Melanoma	3/4	50%
Small Cell Lung Carcinoma	3/4	75%
Renal Carcinoma	1/3	33%
Ovarian Carcinoma	0/5	0%
Pancreatic Carcinoma	0/5	0%

*Using mAb 8B6 as described in Material and Methods.

### Binding of mAb 8B6 to GD2-expressing cell line

Several groups have shown that tumor cells that express GD2 ganglioside also express *O*AcGD2 [18], [19], [20]. The extent to which mAb 8B6 reacted with several types of human and mouse tumor cell lines was determined by flow cytometry analysis. All cell types that expressed GD2 ganglioside were found to also express *O*AcGD2. These data were confirmed by analysis of the tumor cell ganglioside content by immuno-thin-layer chromatography using mAbs 8B6 and 14G2a (data not shown). It should be noted that in these experiments, mAb 14G2a showed a slight cross reactivity against *O*AcGD2 in agreement with a previous report [18]. We next calculated the number of *O*AcGD2 molecules and of 14G2a's epitopes present at the cell surface by Scatchard analysis using ^125^I-labeled mAb 8B6 and ^125^I-labeled mAb 14G2a respectively. As summarized in Table 3, cell lines revealed different levels in the number of mAb binding sites. EL4, NXS2 and IMR32 cell lines expressed large amounts of *O*AcGD2 with mAb 8B6 antibody site numbers ranging from 0.5×10^6^ to 5.5×10^6^ sites/cell. The amount of 14G2a binding sites was found to be comparable with 0.7×10^6^ sites/EL4 cell, 5.3×10^6^ sites/NXS2 cell and 12×10^6^ sites/IMR32 cell. The H82 cell line showed an intermediary binding site number of 0.1×10^6^ sites/cell for *O*AcGD2 and GD2. The OVCAR-3 and U87MG cells showed less than 5×10^4^ sites/cell, whereas Neuro2a cells express neither *O*AcGD2 nor GD2 ganglioside. These data confirm the overexpression at the cell surface of ganglioside *O*AcGD2 on numerous GD2-expressing tumor cell lines. The affinity constants of mAbs 8B6 and 14G2a were also calculated. The K*D* value of mAb 8B6 for *O*AcGD2 was found to be 32 nM and of mAb 14G2a's K*D* value for GD2 was calculated to be 49 nM.

**Table 3:** Binding properties of mAbs 8B6 and 14G2a on tumor cell lines*.

Origin	Cell line	*O*AcGD2 expression (×10^6^ /cell)	GD2 expression (×10^6^ /cell)
**Human**			
Neuroblastoma	IMR32	5.5	12.6
Small cell lung carcinoma	NCI-H69	0.16	0.19
Ovarina carcinoma	OVCAR-3	0.02	0.04
**Mouse**			
T lymphoma	EL4	0.53	0.74
Neuroblastoma	NXS2	0.52	1.3
	Neuro 2a	0	0

*Determined by Scatchard analyses using ^125^I-labelled mAb 8B6 or ^125^I-labelled mAb 14G2a.

### Reduction of tumor cell viability in vitro

**Figure 2:**
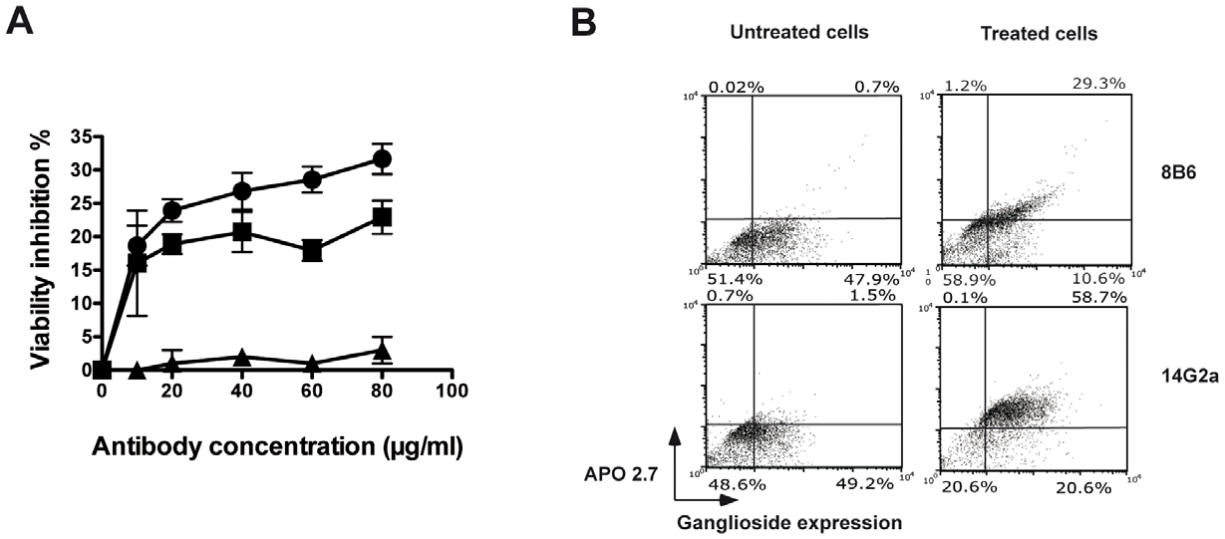
Antibody 8B6 and mAb 14G2a each induce viability inhibition and apoptosis of EL4 cells. (A), EL4 cell line was treated for 24 hours with various concentrations of mAb 8B6 (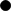), mAb 14G2a (▪) and a control 4F6 antibody (▴). Viability was assessed as described in « Materials and Methods » by adding the methylthiazole tetrazolium salt during 4 hours (MTT assay). Optical density was recorded at 570 nm. The data are presented as the mean ± SD for three independent experiments, each in triplicate. (B), On the treated right column, EL4 cells were exposed to either 50 µg/mL of mAb 8B6 or mAb 14G2a for 24 hours and then double stained with fluorescein-isothiocyanate-conjugated F(ab')_2_ fragments of goat anti-mouse IgG (H+L). After permeabilization, cells were stained with Apo2.7-PE antibody as described in ‘ Materials and Methods’. The percentage of double-positive cell in the untreated EL4 tumor cells is indicated in the left column. Numbers in quadrants represent the percentage of cells in each section of the quadrant.

Since *in vitro* cell culture experiments have shown that various anti-GD2 mAbs inhibit tumor cell growth by directly inducing apoptosis [8], [21], [22], we studied whether the mAb 8B6 displayed the same effects on tumor cell viability. To test for the antitumor activity of mAb 8B6, we choose the EL4 cell line because it is tumorigenic in syngeneic immunocompetent C57Bl/6 mice and because it was used previously in many preclinical studies with anti-GD2 mAbs [23]. Cells were incubated with either mAb 8B6 or mAb 14G2a over a period of 72 hours. Cell viability was determined by MTT assay. The control 4F6 antibody and the Neuro 2a cell line were included to ensure that the observed result was antigen-specific. The inhibitory effect on EL4 cell viability of both mAbs 8B6 and 14G2a was dose- and time-dependant (data not shown) and became statistically significant at 24 hours post treatment at 20 µg/ml (p<0.01) when compared to mAb 4F6-treated cells (Fig 2A). As expected, neither mAb 8B6 nor mAb 14G2a suppressed the growth of the antigen-negative Neuro 2a cell line (data not shown). Overall, these results show the ability of mAb 8B6 to inhibit tumor cell viability, independently of immunological mechanisms such as CDC and ADCC.

### Antibody induced tumor cells apoptosis

To test the ability of both mAbs to induce programmed cell death, we stained antigen-expressing tumor cells with Apo2.7 antibody, followed by flow cytometry analyses, and compared results to control 4F6 antibody-treated cells. Apoptotic cells were detected by flow cytometric analysis after staining bound mAbs to either GD2 or *O*AcGD2 with a FITC-conjugated goat anti-mouse IgG F(ab')_2_ fragment goat anti-mouse IgG. This analysis differentiates the antigen expressing cells from the apoptotic one. As shown in Fig. 2B, addition of either mAb 8B6 or mAb 14G2a to the EL4 culture medium resulted in an increased percentage of double-positive cells. Encouragingly, the effects of mAb 8B6 and mAb 14G2a were comparable with about 75% of antigen-positive cells undergoing apoptosis. The above finding was confirmed by fluorescence microscopy of EL4 cells after Hoechst 33342 staining. Microscopic analysis clearly showed bright nuclear staining and highly condensed nuclei with condensed and fragmented chromatin induced by treatment with either mAb 8B6 or mAb 14G2a (data not shown). These results show the ability of mAb 8B6 to induce apoptosis in *O*AcGD2–expressing cell lines similarly to mAb 14G2a specific for GD2.

### Antibody induced ADCC and CDC

**Figure 3:**
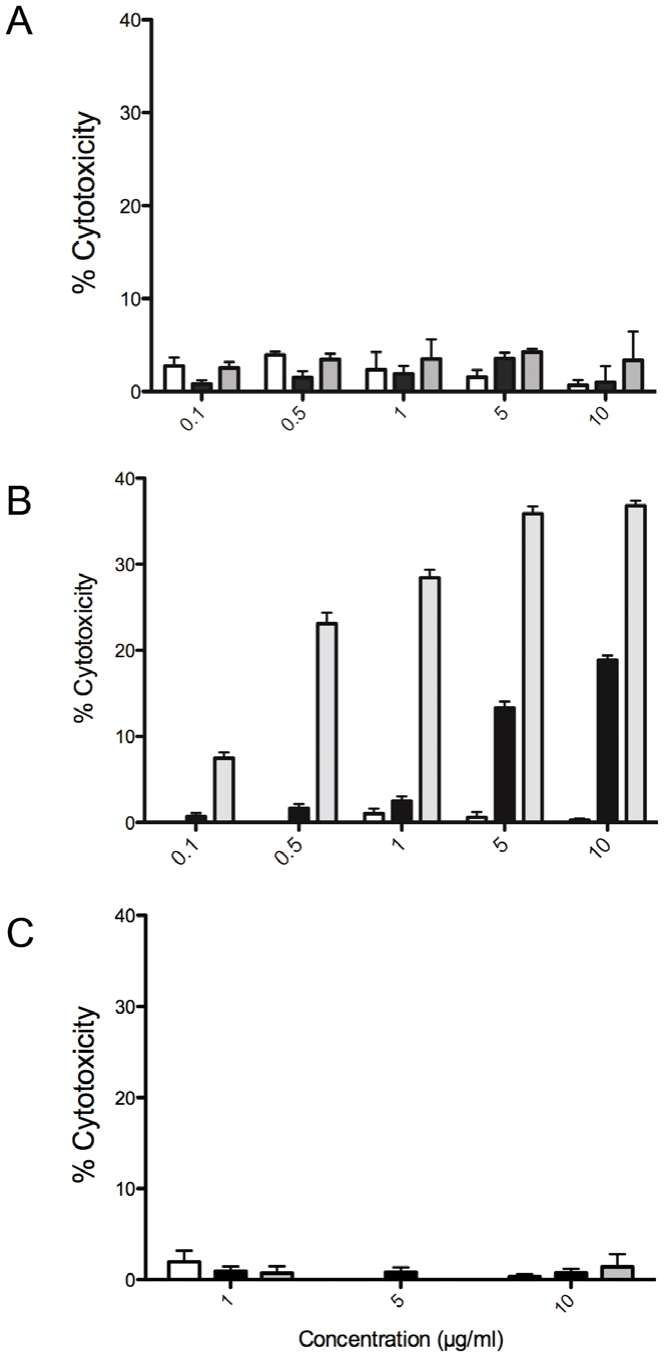
Activation of complement by mAb 8B6 and mAb 14G2a. Complement-dependent specific lysis was determined for the EL4 cell line (A), the NXS2 cell line (B), and the *O*AcGD2/GD2-negative Neuro 2A cells (C) as described in Material and Methods. Empty columns, irrelevant antibody; black columns, mAb 8B6; grey columns, mAb 14G2a.

The capacity mAb 8B6 or mAb 14G2a to induce CDC and ADCC with EL4 cells in the presence of A-LACK cells and complement from C56BL/6 mice was next evaluated. For CDC assays, the *O*AcGD2/GD2-expressing target cells were incubated with mAb 14G2a in the presence of diluted mouse serum as complement. Cell death was assessed by the addition of the viability probe propidium iodide. As shown in Fig. 3A, neither mAb 8B6 nor mAb 14G2a mediated CDC on EL4 cells in the presence of C57BL/6 mouse complement (Fig. 3A). This result was not surprising because EL4 cell express the rodent inhibitor of complement activation Crry that protects EL4 cells from complement deposition [10]. Therefore, mAb 8B6 CDC testing was performed with the NXS2 cells. By contrast to EL4 cells, an efficient CDC was observed when NXS2 cells were used (Fig. 3B). Specific lysis achieved maximum values of 36.8±1.4% when mAb 14G2a was used but only 18.8±1.9% with mAb 8B6. Cytotoxicity correlated directly with the concentration of antibody. Specificity was demonstrated by comparing the CDC results of mAb 8B6 and mAb 14G2a with non-specific controls using anti-GD3 mAb, which showed only background lysis. Specificity was also demonstrated with the *O*AcGD2/GD2 negative Neuro 2A cells where both mAb 8B6 and 14G2a were ineffective in activating complement dependant cytotoxicity (Fig. 3C).

For ADCC reactions, the A-LAK cells generated from C57BL/6 mice were composed by ∼80% of CD3-negative, NK1.1-positive NK cells and ∼20% of CD3-positive, NK1.1-positive NKT cells and the two populations expressed the Fcgamma RIII receptor (data not shown). The target cells were labeled with a membrane dye, PKH-26, to allow discrimination when incubated with effector cells and antibody. Post-incubation, cell death within the PKH-26 + target cell population was detected by the addition of the viability probe TP3.

**Figure 4:**
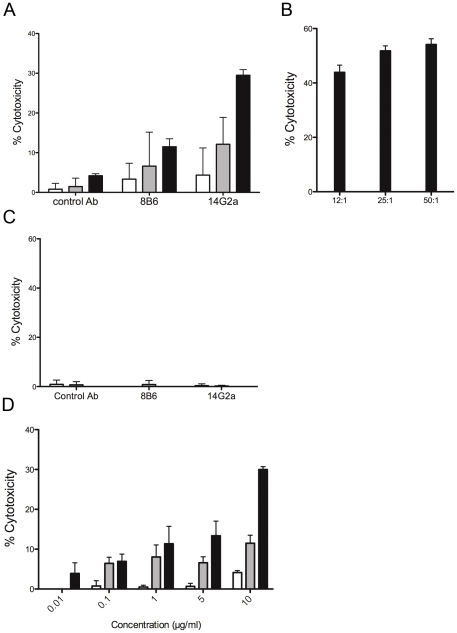
ADCC of mAb 8B6 and mAb 14G2a. (A) The A-LAK killer activity with mAb 8B6 and mAb 14G2a with EL4 target cells at the E/T ratio 12 to 1 (empty columns), 25 to 1 (grey columns), and at the E/T ratio 50 to 1 (black columns) as described in Material and Methods. (B) The A-LAK killer efficiency in the ADCC assays was tested using the sensitive YAC-1 target cells. (C) ADCC activity with mAb 8B6 and mAb 14G2a against the *O*AcGD2/GD2-negative Neuro 2A cells used as a negative control, at the E/T ratio 12 to 1 (empty columns), 25 to 1 (grey columns), and at the E/T ratio 50 to 1 (black columns). (D) ADCC mediated by mAb 8B6 (grey column) and mAb 14G2a (black column) with EL4 target cells at varying antibody concentrations. The results were compared to the effect of equal amount of irrelevant antibody used as a negative control (*n* = 3).

ADCC was observed with both mAb 8B6 and mAb 14G2a against *O*AcGD2/GD2-expressing target cells when C57BL/6 mice A-LACK cells were used (Fig. 4A). However, mAb 14G2a induced a more effective ADCC than mAb 8B6 with specific lysis achieved maximum values of 29.5±1.4% with mAb 14G2a, but only 11.5±2% with mAb 8B6 (Fig. 4A). This result was also unsurprising since earlier works reported that mouse IgG3 mAbs against ganglioside antigens are poorly effective *in vitro* in inducing CDC and ADCC with mouse complement and mouse effector cells [24], [25]. On the other hand, EL4 cells were efficiently killed when human NK effector cells were used with a maximum value of specific lysis of 30% (Fig. S5). Cytotoxicity correlated directly with the E/T ratio (Fig. 4A) and the antibody concentration (Fig. 4D). The A-LAK killer efficiency was demonstrated with the sensitive YAK-1 cells where specific lysis reached maximum value of 51.4±1% (Fig. 4B). Specificity was demonstrated by comparing the ADCC results of mAb 8B6 and mAb 14G2a with non-specific controls using anti-GD3 mAb, which showed only background lysis (Fig. 4A). Specificity was also demonstrated with the *O*AcGD2/GD2 negative Neuro 2A cells where both mAb 8B6 and 14G2a were ineffective in activating complement dependant cytotoxicity (Fig. 4C).

### Analysis of in vivo antibody antitumor activity

**Figure 5:**
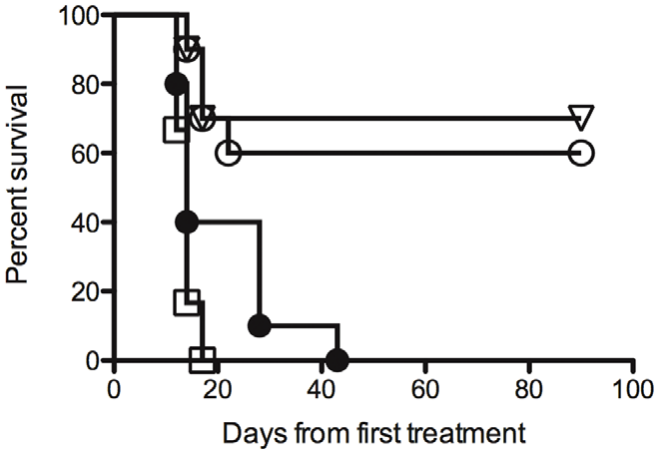
Survival of C57BL/6 mice inoculated with EL4 cells treated with either 8B6 or 14G2a mAb. Mice (*n* = 10) were inoculated with 10^4^ EL4 cells subcutaneously and then treated with either PBS or 70 µg of each antibody, twice weekly for 3 weeks. PBS (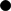), mAb 8B6 (○), mAb 14G2a (▿), control 4F6 antibody (□).

We next evaluated whether mAb 8B6 could be used to treat transplanted tumor. To determine if the *O*AcGD2 mAb 8B6 can suppress tumor formation, we injected the EL4 syngeneic T-lymphoma to C57BL/6 mice. Twenty four hours after tumor inoculation, groups of 10 mice received 70 µg per dose of mAb 8B6, mAb 14G2a, irrelevant mAb, or PBS twice a week for 3 weeks. After tumor injection, we monitored the tumor volume and the health of the mice. We measured the tumors biweekly and euthanized the mice when their tumor size exceeded 2,000 mm^3^. The survival curves were plotted according to the Kaplan-Meier method and compared using the log-rank test. The results are summarized in Fig. 5. In the vehicle-treated group and in the irrelevant antibody-treated group all animals developed large tumors that were detected within 2 weeks after the initial inoculation, and had died by day 42. In contrast, 60% of the mice that had received mAb 8B6 were still tumor free after 90 days and were considered cured. In comparison, 70% of the mice were still tumor free in the mAb 14G2a treated group. The effect of mAb 8B6 treatment was not statistically different from that obtained upon treatment with mAb 14G2a (*p*<0.05). The specificity of mAb 8B6 therapy was demonstrated, since treatment with an equivalent amount of non-specific IgG3 antibody was completely ineffective. Notably, this control antibody binds to GD3 ganglioside that is not express by EL4 cells (data not shown).

The anti-neuroblastoma efficacy of mAb 8B6 was also determined in the NXS2 mouse neuroblastoma experimental liver metastasis model developped by lode *et al*.[26] (Fig. S6). The treatment of mice (n = 9) at the dose of 100 µg mAb 8B6/day for 5 days was dramatically effective in reducing neuroblastoma liver metastasis, as indicated by a decrease of the liver weight from 2.8 g±0.8 g (PBS) to 1.2 g±0.8 g (mAb 8B6 treated mice) and 1.1 g±0.2 g mAb 14G2a (p<0.001) (Fig. S5.A). The latter two values were not statistically different from those found in healthy control animals (p>0.1). The effect of mAb 8B6 treatment was not statistically different from that obtained upon treatment with mAb 14G2a (p>0.5). The specificity of mAb 8B6 therapy was again demonstrated, since treatment with an equivalent amount of non-specific antibody was completely ineffective. Taken together, our results show the potential therapeutic efficacy of mAb 8B6 for the treatment of GD2/*O*AcGD2-expressing tumors.

## Discussion

The most striking result of this study is that mAb 8B6, a mouse monoclonal antibody specific for OAcGD2 that does not bind GD2, did not show any reactivity at all with peripheral nerves. By contrast, the anti-GD2 antibody 14G2a that was used as a positive control stained peripheral nerve fibers, which are known to express GD2 [6]. In these study, we selected an immunoperoxydase assay performed on frozen tissue sections according to the FDA guidelines [27]. In the absence of characterization of the *O*-acetyl-transferase, the enzyme responsible for the biosynthesis of *O*-acetylated ganglioside (for review see [28]), the results suggest that GD2 is differentially acetylated in normal and tumor tissue and that normal tissues expressing GD2 may not express OAcGD2, as is known for GD3 (for a review see [29]). Antibody 8B6 did not stain or stain very weakly the normal tissues that must be tested before clinical tested, as required by the FDA, with the exception of lymph node germinal centers. This exception may be considered as a positive control for the ICH study since GD2 has been shown to be expressed in lymph node germinal centers [7].

As mentioned earlier, the therapeutic use of anti-GD2 mAbs is associated with important neurotoxic effects in patients. The proposed cause of this dose-limiting toxicity is the binding of anti-GD2 antibodies to GD2 expressed on normal nerve cells followed by complement deposition on the nerve cell surface [6]. Hence, our data suggest that mAbs specific for *O*AcGD2 should be less toxic because they do not bind to peripheral nerves, thereby allowing dose escalation of antibodies. Some other side effects observed in patients after anti-GD2 mAb infusions included hematopoietic suppression [30] and a syndrome of inappropriate antidiuretic hormone [6]. The immune recognition of GD2 on mesenchymal stromal cells in the marrow microenvironment was suggested to underlie the hematopoietic suppression and anti-GD2 mAb cross-reactivity with the posterior lobe of the pituitary gland is believed to modulate the secretion of antidiuretic hormone resulting in the induction of the syndrome of inappropriate antidiuretic hormone secretion[6]. Interestingly, mAb 8B6 did not show any binding to mesenchymal stromal cells in the bone narrow nor to the posterior lobe of the pituitary gland. We also examined the immunohistochemical *O*AcGD2 localization in a number of malignant tissues and found that mAb 8B6 showed strong reactivity with neuroectodermic tumor biopsy tissues, such as melanoma and neuroblastoma similar to previous investigations [18], [19].

We further demonstrated the high expression of *O*AcGD2 at the tumor cell surface by Scatchard analysis *in vitro*. In the 7 tested cell lines, the average of sites/cell ranged from 50,000 sites/cell up to 5×10^6^ sites/cell. The expression level of *O*AcGD2 in cell lines was first described based on extraction and thin layer chromatography (TLC) or immune TLC [18], [19]. These methods cannot discriminate the membrane from intracellular *O*AcGD2 cell content. Importantly, our data shows that the amount of *O*AcGD2 molecules present at the cell surface is comparable, though lower, to that of mAb 14G2a epitope. However, the number of GD2 molecules calculated here may be overestimated as previous reports suggested that mAb 14G2a cross-reacts with *O*AcGD2 [18]. In agreement with this earlier study, we did see a slight cross-reactivity of mAb 14G2a against *O*AcGD2 in immuno-TLC experiments (data not shown). Scatchard analysis further showed that mAb 8B6 and mAb 14G2a displayed equivalent binding affinities for their respective epitopes that were within the range anti-GD2 antibodies [21], [24], [31], [32]. *O*AcGD2 expression was confirmed on all of the 12 neuroblastoma tumor sections tested. This is consistent with a previous study reported by Ye and Cheung [19] that *O*AcGD2 is a naturally occurring GD2 derivative in neuroblastoma tumors.

We also showed that *O*AcGD2 is a pro-apoptotic constituent activated on binding with hostile antibodies. Despite its high expression at the tumor cell surface, the biological role of *O*AcGD2 has yet not been studied so far. The results presented here demonstrate that *O*AcGD2 behaves very similarly to GD2 in mediating apoptosis in the GD2/*O*AcGD2-expressing tumor cells and contrast with previous work on *O*AcGD3 functions in tumor cell biology. *O*-Acetylation of GD3 prevents apoptosis in lymphoid cells induced by various agents—including GD3 itself—and therefore sustains tumor progression [33], [34]. While *O*AcGD2 can transmit signals resulting in apoptosis, the precise mechanisms induced by the binding of *O*AcGD2 antibody to *O*AcGD2-expressing tumor cells leading to apoptosis requires further investigation. In the case of GD2, initial indications suggest that anti-GD2 mAbs induce apoptosis of SCLC cells by interfering with the association of GD2 ganglioside to ß1-integrin and focal adhesion kinase [35]. The *O*AcGD2-triggered apoptosis may follow the same pathway, for the ß1-integrin binding site to GD2 might exclude the outer sialic acid that becomes *O*-acetylated in *O*AcGD2. However, since the addition of an *O*-acetyl ester to sialic acid changes its structural properties and affects its binding specificity, the association between *O*AcGD2 and integrin remains open.

Finally, we reported for the first time that passive immunotherapy with mAb 8B6 to *O*AcGD2 is effective in suppressing the growth of *O*AcGD2-expressing tumor in two animal models. We used the EL4 murine lymphoma that is syngeneic in C57BL/6 mice and the murine NXS2 neuroblastoma that is syngeneic in A/J mice. These two cell lines express GD2 and as demonstrated here *O*AcGD2. These two cell lines were retained because they were previously used in the anti-GD2 antibodies preclinical setting [9], [23]. Our results further demonstrate that the anti-tumor efficacy of mAb 8B6 is comparable to that of anti-GD2 mAb 14G2a, which has undergone clinical evaluation with positive results [14], [15], [36]. Antibody 14G2a is also the parental mouse antibody of human-mouse chimeric ch14.18 that has recently shown clinical efficacy in a phase III trial [4]. ADCC has been proposed as the most critical effector function *in vivo* for mAbs against GD2 gangliosides [9], [10]. In our experiments we used mAb 8B6 which is a mouse IgG3. Despite past controversy about the presence or the absence of a mouse IgG3 Fc-receptor, this isotype is now well known for its inability to promote ADCC with mouse effector cells both *in vitro* and as *in vivo*, as it barely binds to mouse FcgammaRs [37], [38]. Then, as expected, mAb 8B6 failed to show any ADCC activity in the presence of mouse spleen cells, but was effective in directing ADCC against EL4 cell line with human NK cells. Moreover, our data indicate here that the EL4 cells used in our experiments were resistant to mouse complement. This finding is in agreement with Imai *et al*. [10], who have reported that EL4 cells express the rodent inhibitor of complement activation Crry that inhibits complement activation at the C3 activation step, protecting EL4 cells from complement deposition and lysis [10]. Interestingly, the absence of sensitivity to complement does not appear to affect the anti-tumor effect of anti-GD2 mouse IgG mAbs except at low antibody concentration *in vivo* [10]. The absence of Fc-directed CDC/ADCC functions requirement for anti-GD2 mAb anti-tumor efficacy in vivo was also suggested by Mujoo *et al*. [24] who studied the anti-tumor properties of mouse IgG3 mAb 14.18 and its isotype switch variants. In their study, mAb 14.18 was demonstrated to be as effective as mAb 14G2a in suppressing neuroblastoma growth in mice and no correlation could be drawn between the in vivo anti-tumor effects of these antibodies and their in vitro functions such as directing ADCC and CDC [24].

In as much as mAb 8B6 directs neither ADCC nor CDC with EL4 cells when mouse immune effectors are used, the mechanism whereby it mediates *in vivo* suppression of tumor growth in this model is also most likely to involve its pro-apoptotic properties. Although the mechanism remains to be elucidated in vivo, from a clinical standpoint, the apoptosis inducing activity of mAb 8B6 specific for *O*AcGD2 evidenced here seems very promising when applied to cancer therapy. Additional biological processes after antibody binding to tumor cells such as CDC and ADCC have been demonstrated *in vivo*. However, pro-apoptotic properties without immune effector mechanisms may be important in the treatment of tumors that have evolved complex mechanisms to protect themselves from ADCC and CDC. We are currently generating a human-mouse chimeric anti-*O*AcGD2 IgG1 form mAb 8B6 in order to better define the role of immune effector mechanisms involved in the anti-tumor properties of anti-*O*AcGD2 antibodies. Whatever the effector mechanism(s) involved, our results strongly support the clinical use of anti-*O*AcGD2 mAbs. In contrast to anti-GD2 therapeutic antibodies, they may offer an effective treatment option with reduced adverse side effects, thereby allowing dose escalation of antibodies, and the development of more potent immunoconjugates such as immunotoxines or radio-conjugates and immunocytokines. In addition to neuroblastoma, melanoma, *O*AcGD2 is also expressed on osteosarcomas, ovarian carcinomas [18], [19], [20] and, as reported here, on small cell lung carcinomas, suggesting that anti-*O*AcGD2 immunotherapy is applicable to all these types of cancers. In all, *O*AcGD2-expressing diseases account for ∼7% of all death in the US [39].

## Materials and Methods

### Cell culture

Cell lines were obtained from the American Type Culture Collection (ATCC), except for the mouse neuroblastoma NXS2 cell line which was a courtesy of Dr. H. N. Lode (*Universitätsklinikum Greifswald*, Greifswald, Germany) and the YAC-1 cells (courtesy of B. Clemenceau, Inserm U. 892, Nantes, France). Murine T-lymphoma EL4 cells were grown at 37°C in 5% CO_2_ in DMEM with 10% heat-inactivated FCS, 100 units/mL penicillin, and 100 µg/mL streptomycin. Human neuroblastoma IMR32, human glioma U87MG, human small cell lung cancer H82, human ovarian adenocarcinoma OVCAR-3, murine lymphomaYAC-1, and mouse neuroblastoma Neuro 2a cell lines were grown at 37°C in 5% CO_2_ in RPMI 1640 with 10% heat-inactivated FCS, 100 units/mL penicillin, and 100 µg/mL streptomycin. The mouse neuroblastoma NXS2 cell line was grown at 37°C in 5% CO_2_ in DMEN with 10% heat-inactivated FCS, 100 units/mL penicillin, and 100 µg/mL

### Antibodies and serum

Anti-*O*AcGD2 mAb 8B6 (mouse IgG3, kappa) was obtained previously in our laboratory [17]. An IgG3 antibody specific to GD3 ganglioside (clone 4F6) was used as a negative control (generously provided by Dr. Jacques Portoukalian, Department of Dermatology, Edouard Herriot Hospital, University of Lyon, France) [17]. Monoclonal antibodies 8B6 and 4F6 were purified from hybridoma supernatants using Protein A affinity chromatography. The purity of mAb preparations was verified by SDS-PAGE analysis. Anti-disialoganglioside GD2 mAb 14G2a was purchased from BD Biosciences (Franklin Lakes, NJ). The mouse IgG3 mAb negative control from AbD serotec (Oxford, UK) was used as a negative control in some experiments.

### Human tissues reactivity of mAb 8B6

Portions of fresh peripheral nerves and malignant tissues were provided by the Pathology department at the *Centre Hospitalier Universitaire*, Nantes, France. Eight fragments of neuroblastoma samples were provided by the Children's Hospital Oakland Research Institute Tissue Bank (Oakland, CA, USA). Fresh tissue specimens were embedded in Tissue Tek-II O.C.T. (Miles, Naperville, IL), snap frozen in isopentane in liquid nitrogen, and stored at −70° C. Ten micrometer-sections were cut, fixed in acetone, and stained with mAb 8B6 and mAb 14G2a respectively for 1 hour. After rinsing, the APAAP complex (Dako) was applied. Then, the bound mAb was detected with ImmPACT DAB chromogen substrate solution (Vector Laboratories, Bulingore, CA), which was used to produce a brown deposit. The mouse IgG3 negative control reagent (AbD serotec) was used as a negative control, and neuroblastomas were used as positive control. The concentration of 2.5 µg/mL was selected for the study. Slides were counter-stained with hematoxylin before immunocytological evaluation. Staining was graded as positive or negative according to the presence or absence of immunoreactivity, respectively.

The potential cross-reactivity of mAb 8B6 was also evaluated by using an indirect immunoperoxydase assay on a panel of 32 tissues from unrelated donors according to the FDA guidelines [27] and on a panel of melanomas, small cell lung carcinomas, renal carcinomas, ovarian carcinomas and pancreatic carcinomas. This study was performed independently by Lifespan Biosciences (Seattle, WA, USA). Tissues were stained with positive control antibodies specific for CD31 and for vimentin to ensure that the tissue antigens were preserved and accessible for immunohistochemical analysis. Only tissues that were positive for CD31 and vimentin staining were selected for mAb 8B6 immunoreactivity. Anti-*O*AcGD2 8B6 mAb was used as the primary antibody, and the principal detection system consisted of a Vector anti-mouse secondary (Vector Laboratories), and a Vector ABC-AP kit (Vector Laboratories) with a Vector Red substrate kit (Vector laboratories), which was used to produce a fuchsia-colored deposit. The mouse IgG3 negative control reagent (AbD serotec) was used as a negative control. Slides were imaged with a DVC 1310C digital camera coupled to a Nikon microscope. Images were stored as TIFF files with Adobe Photoshop.

### Flow-cytometric analysis

Cell surface OAcGD2- and GD2-expression on tumor cell lines was assessed by indirect immunofluorescence. Tumor cells (5×105 cells) were incubated with either mAb 8B6, mAb 14G2a or mAb 4F6 (control antibody), at 10 µg/mL for 30 minutes at 4°C in 0.1% BSA-PBS. After reaction with the fluorescein-isothiocyanate-conjugated F(ab')_2_ fragment of goat anti-mouse IgG (H+L) as a second antibody (Jackson, Immunoresearch, Soham, UK) for 30 min at 4°C, cell fluorescence was analyzed using a FACScan flow cytometer (BD Biosciences, San Jose, CA) and CellQuest software (BD Biosciences).

### Scatchard analysis

Monoclonal antibodies were labeled with iodine-125 (Perkin Elmer, Billerica, MA) using the iodogen method and were purified on a Sephadex PD10 column (Pharmacia Biotech, Uppsala, Sweden). Binding assays were performed as previously described [40]. Serial dilutions of labeled antibody were incubated for 1 hour at 4°C with 1×10^6^ cells. Cell-bound radioactivity was separated from free antibody by centrifugation through a dibutyl phthalate oil cushion in microfuge tubes. Cell Pellets and supernatant activities were then separately measured using a gamma counter (Wallac, Finland). Nonspecific binding, defined as the cell-bound ^125^I-labeled antibody in the presence of a 100-fold excess of unlabelled antibody, was subtracted at each concentration of labeled antibody. The binding data were analyzed using the Prism software (GraphPad Prism Software, La Jolla, CA) according to a one-site equilibrium binding equation.

### Cell growth inhibition

Cell viability was measured using the MTT assay [41]. Briefly, 1×10^5^ cells suspended in 200 µL were incubated for 24 hours at 37°C, 5% CO_2_. Monoclonal antibodies were diluted in 100 µL of specific medium and added to each well of 96-well microtiter plates to give the final concentrations of 80, 60, 40, 20 and 10 µg/mL. After incubation for 24 hours at 37°C, 5% CO_2_, 10 µL of methylthiazole tetrazolium salt stock solution (5 mg/mL, Sigma Aldrich, Saint Louis, MO) were added to each well and the plates incubated at 37°C for 4 hours. Then, 100 µL of 10% Triton X-100 were added and the plates incubated for 10 minutes at 37°C for color development. Optical density was recorded at 570 nm on a Multiskan reader (Thermo Electron, Walthman, MA).

### Apoptosis induction

Cells (1×10^5^ cells) were plated in 96-well microplates during 24 hours at 37°C 5% CO_2_ and then treated for 24 hours at 37°C with 50 µg/mL of mAb 8B6, mAb 14G2a and control mAb, respectively. After reaction, cells were incubated with fluorescein-isothiocyanate-conjugated F(ab')_2_ fragments of goat anti-mouse IgG (Jackson) as described above. They were then permeabilized with 100 µg/mL digitonin and stained for 30 min with Apo2.7-PE conjugated antibody and analyzed by flow cytometry using a FACScan flow cytometer and Flowjo6 software (BD Biosciences). Apoptotic cells were analyzed on Apo2.7 (FL2) histograms and compared to *O*AcGD2 positive and negative cell populations determined by FITC staining (FL1).

### Complement dependent cytotoxicity (CDC)

Aliquots of tumor cells (104 cells) were incubated with 80 µL of antibody at various concentration in the presence of 20 µL of mouse-serum as complement source for 1 hour at 37°C. Cytotoxicity was determined within the tumor cell population after addition of the viability probe propidium iodide (PI) using a FACScan flow cytometer (BD Biosciences, San Jose, CA) and the CellQuest software (BD Biosciences). The percentage of specific lysis was calculated as: 100×(nonviable PI+ tumor cells)/(non viable PI+ tumor cells + viable tumor cells).

### Antibody-dependent cell-mediated cytotoxicity (ADCC)

An ADCC assay was performed as reported previously [42]. Briefly, tumor cells were labeled with membrane dye PKH-26 (Sigma Aldrich) according to the manufacturer's instructions. Aliquots of the labeled cells were distributed into a 96-well microtiter plate (1×104 cells /100 µL) were incubated with 25 µL of antibodies in 96-well microtiter plates. Adherent lymphokine-activated killer (A-LACK) cells isolated from spleen cells of C57BL/6 mice were used as effector cells [43]. Fifty µL of effector cell suspension at the indicated effector-to-target (E/T ratio) were added to the tumor cells and incubated for 4 hours at 37°C. Cell death within the PKH-26+ target cell population was then assessed by the addition of TO-PRO-3 iodide (TP3) (Invitrogen) [44]. The double-positive dead target cell population was detected by flow cytometry analysis using a FACScan flow cytometer (BD Biosciences) and the CellQuest software (BD Biosciences). The percentage of specific lysis was calculated as: 100×(nonviable TP3+, PKH26+ double-positive target cells)/(non viable double-positive target cells + viable PKH26+ target cells). The lysis of the NK-sensitive mouse T cell lymphoma YAC-1 was used as an indicator of A-LAK activity [45].

### Murine tumor model

C57BL/6 mice, aged between 8 and 12 weeks, were purchased from Charles River (L'Arbresle, France). Mice were housed at the animal facility of Inserm U892 (Nantes, France). This facility is approved by the French Association for Accreditation of Animal Care Laboratories and is maintained in accordance with the regulations and standards of Inserm Institute and the French Department of Agriculture. The EL4 cell line is a syngeneic murine lymphoma in C57BL/6 mice that was previously used by Imai *et al*. as a target for anti-GD2 antibodies [10]. Mice were injected subcutaneously with EL4 cells (1×10^4^ cells) in the right flank. Twenty four hours after tumor inoculation, groups of 10 mice received 70 µg per dose of 8B6 mAb, 14G2a mAb, control IgG3 mAb, or PBS twice a week for 3 weeks. Tumor volume and the health of the mice were monitored. After grafts became visible, the sizes of tumors were determined three times a week by externally measuring the tumors in two dimensions. Tumor volume was calculated according to the equation: *V*  =  (*L*×*W* ^2^) ×0.5, where *L* is the length and *W* the width of a tumor [46]. For ethical considerations, mice had to be euthanized once tumor volume had reached 2,000 mm^3^, which was considered the end point for each individual mouse.

### Statistical analysis

Statistical analysis was performed using Prism software (GraphPad Prism Software). Data are shown as mean ± standard error. Differences between un-treated and treated groups in the *in vitro* experiences were analyzed by Student' *t* test with significance at *p*<0.05.

## Supporting Information

Figure S1 **In Fig. S1, five other representative examples of peripheral nerve stained by either anti-GD2 mAb 14G2a (1) or anti-** ***O*** **AcGD2 mAb 8B6 (2) are shown.** While positive staining of the myelin sheaths—evidenced by brown coloration—is found in all sample stained with anti-GD2 mAb 14G2a, no binding of anti-*O*AcGD2 mAb 8B6 is detected. Scale bar  = 50 µm.
(TIF)

Figure S2 **The synopsis of mAb 8B6 cross-reactivity with human normal tissues is provided in** **Table 1** **.** In Fig. S2, representative results of mAb 8B6 (1) human tissue cross-reactivity with the zona reticularis of the adrenal (A), the germinal center in the lymph node (B), the Purkinje cells in the brain cerebellum (C), the epithelium apical surface of the small intestine (D), the follicular epithelium in the thyroid (E), and the gray matter in the dorsal horns (F) are shown. The tissue cross reactivity study was performed using an immunoperoxydase assay as indicated in the “Material and Methods” section. The mouse IgG3 mAb negative control from AbD serotec (Oxford, UK) was used as a negative control (2). Representative positive staining of mAb 8B6 on melanoma (1) representative negative staining of control IgG3 (2) on melanoma cells (G). Photos are reproduced with the permission of Lifespan Biosciences. Magnification 400×.
(TIF)

Figure S3 **While** **Table 2** **summarize the expression of** ***O*** **AcGD2 on human neuroblastoma tumors, Fig. S4 shows 8 representative examples of** ***O*** **AcGD2 expression on neuroblastomas detected by an immunoperoxydase assay performed with mAb 8B6 as described in the Material and Methods Section.** 1, representative positive staining obtained with anti-GD2 mAb; 2, representative negative staining obtained with the mouse IgG3 mAb negative control (AbD Serotec); 3 to 10, representative diversity patterns from neuroblastoma samples. Antibody 8B6 showed moderate to strong positive staining with all neuroblastomas. The percentage of tumor cells that were positive was 100% and staining was both membranous and cytoplasmic. When the corresponding normal cell types were present in the same cancer samples, they were negative. All samples were also positively stained with mAb 14G2a and negative with irrelevant control mAb (data not shown). Scale bar  = 100 µm.
(TIF)

Figure S4 **While** **Table 2** **summarize the expression of** ***O*** **AcGD2 on human tumor, in Fig. S4 representative example of expression of mAb 8B6 epitope in melanoma cells (A) and small cell lung carcinoma cells (C) are shown.** Skin (B) and lung (D) samples from control patients without cancer showed negative staining in the precursor cell type. The samples were stained with mAb 8B6 using an immunoperoxydase assay performed as described in the “Material and Methods” section. The mouse IgG3 mAb negative control from AbD serotec (Oxford, UK) was used as a negative control (data not shown). Photos are reproduced with the permission of Lifespan Biosciences. Magnification 400×.
(TIF)

Figure S5 **While** **Fig. 4** **shows that mAb 8B6 ADCC activity against mouse EL4 target cells was poorly effective with mouse effector cells, an efficient killer activity of human NK cells with mAb 8B6 is depicted in Fig. S5.** ADCC assays were performed at the E/T ratio 50 to 1 using mAb 8B6 (10 µg/ml, black column). Antibody 8B6 specific antibody-dependent lysis occurred in a dose-dependent fashion. Specificity was demonstrated by comparing the ADCC results of mAb 8B6 with non-specific controls using anti-GD3 mAb (10 µg/ml, empty column), which did not show any significant activity, *n* = 3.
(TIF)

Figure S6 **While** **Fig. 5** **shows the anti-tumor efficacy of mAb 8B6 against EL4 lymphoma cells in C57BL/6 mice, the anti-neuroblastoma activity of mAb 8B6 against established experimental liver metastasis are depicted in Fig. S5.** Mice (*n*  = 9) were inoculated with 0.25×10^6^ NXS2 cells by i.v. injection and then treated 3 days latter with 5 daily i.v. injections of either 100 µg mAb 8B6, 14G2a and irrelevant antibody. Mice were sacrificed 28 days after tumor cell inoculation. (A) The liver weight was determined on fresh specimen. The *y*-axis starts at 0.8 g corresponding to the average normal liver weight. The differences in average liver weights between experimental groups treated with mAb 8B6 and mAb 14G2a and all control groups (PBS, control antibody) was statistically significant (* p<0.001). (B) Representative liver specimen of each experimental group (*n* = 9) are shown. 1, PBS; 2, control IgG3, 3, mAb 8B6; 4, mAb 14G2a. Arrows indicate the location of macroscopic liver metastases.
(TIF)
