# Supplementary figures and images for: A Monoclonal Antibody to O-Acetyl-GD2 Ganglioside and Not to GD2 Shows Potent Anti-Tumor Activity without Peripheral Nervous System Cross-Reactivity
Source: PLoS One. 2011 Sep 22;6(9):e25220. doi: 10.1371/journal.pone.0025220 (PMC3178631; doi:10.1371/journal.pone.0025220)

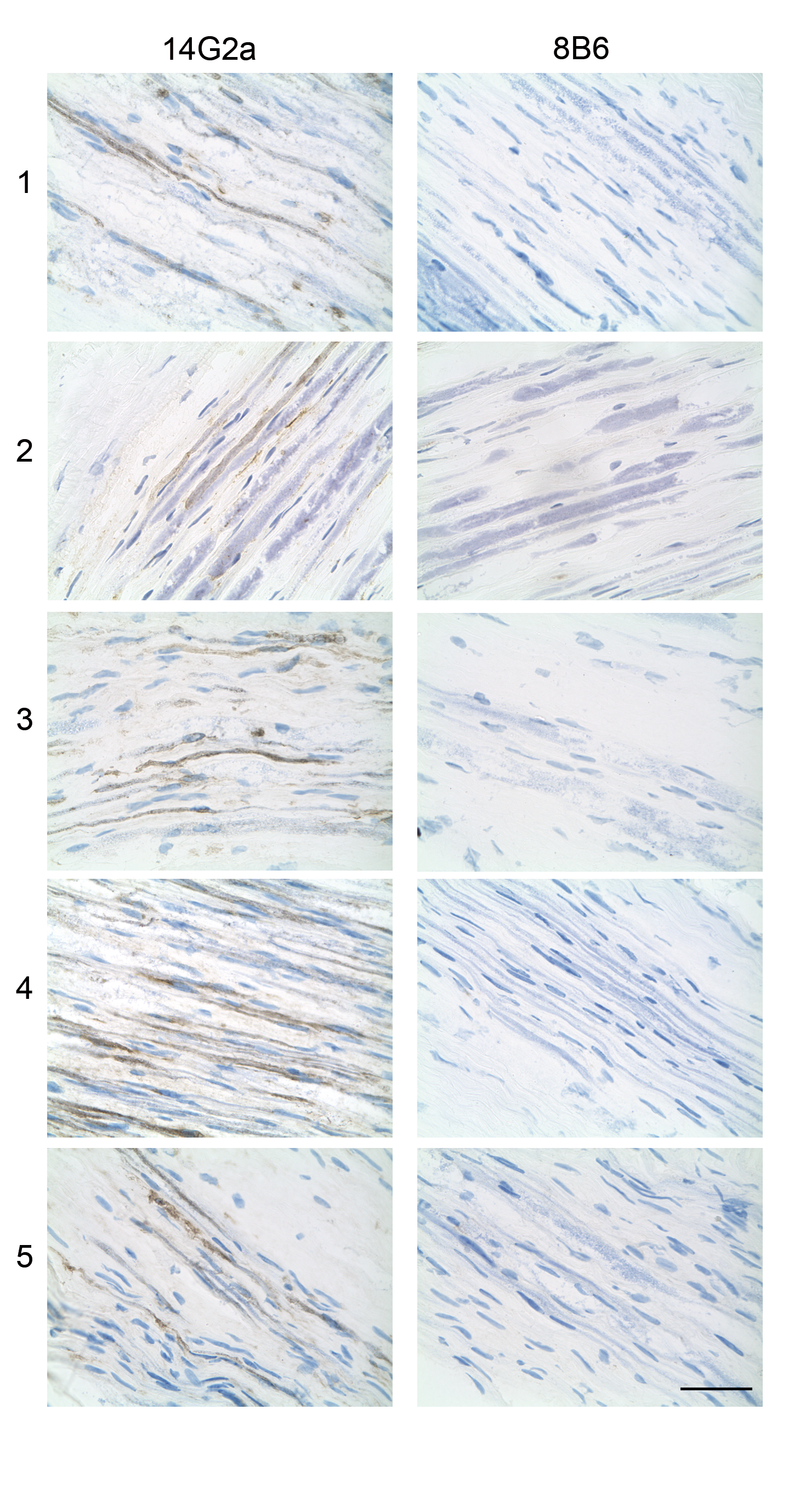

Supplement: Figure S1 — In Fig. S1, five other representative examples of peripheral nerve stained by either anti-GD2 mAb 14G2a (1) or anti- O AcGD2 mAb 8B6 (2) are shown. While positive staining of the myelin sheaths—evidenced by brown coloration—is found in all sample stained with anti-GD2 mAb 14G2a, no binding of anti-OAcGD2 mAb 8B6 is detected. Scale bar = 50 µm. (TIF) [file pone.0025220.s001.tif]

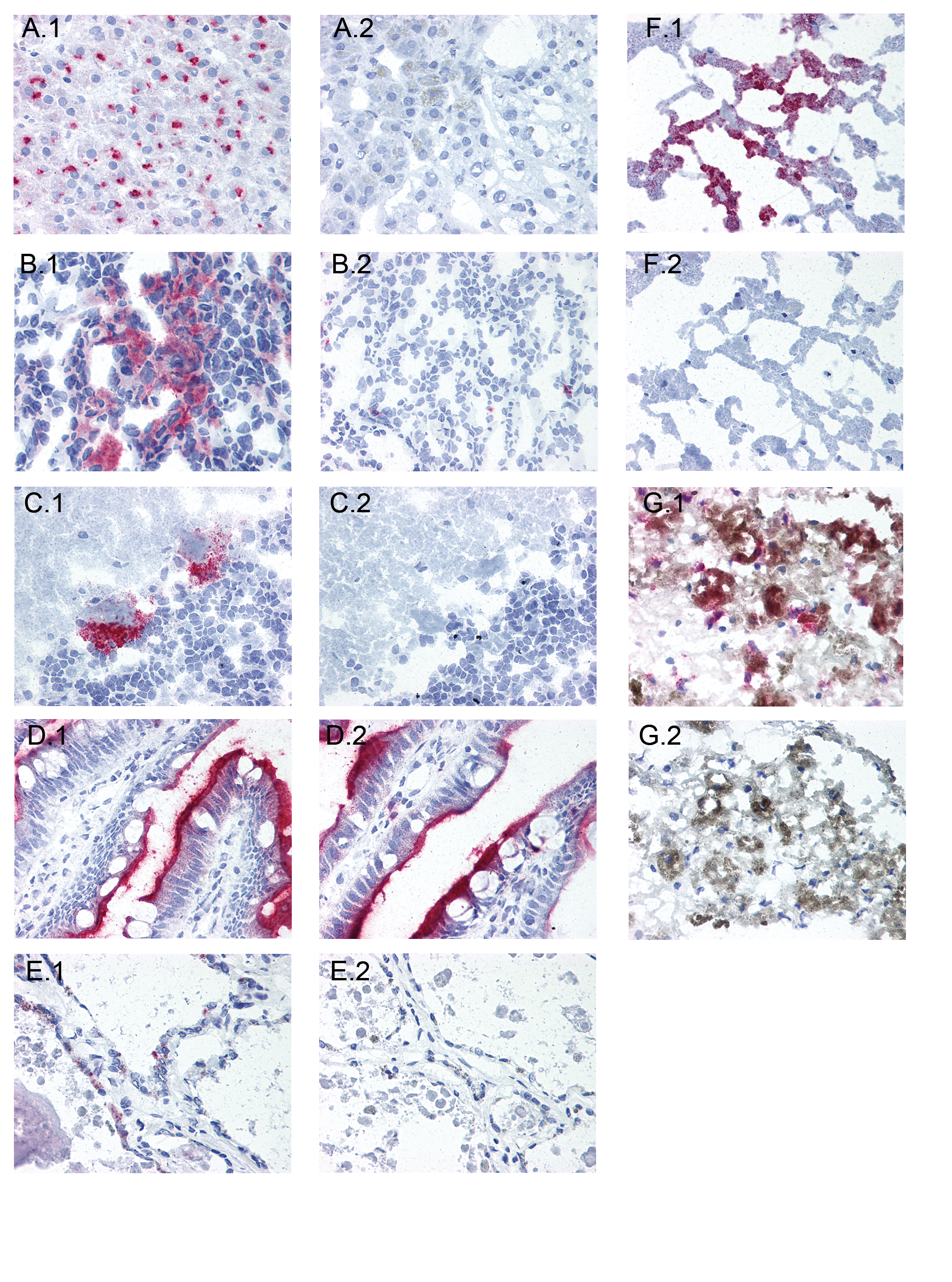

Supplement: Figure S2 — The synopsis of mAb 8B6 cross-reactivity with human normal tissues is provided in Table 1 . In Fig. S2, representative results of mAb 8B6 (1) human tissue cross-reactivity with the zona reticularis of the adrenal (A), the germinal center in the lymph node (B), the Purkinje cells in the brain cerebellum (C), the epithelium apical surface of the small intestine (D), the follicular epithelium in the thyroid (E), and the gray matter in the dorsal horns (F) are shown. The tissue cross reactivity study was performed using an immunoperoxydase assay as indicated in the “Material and Methods” section. The mouse IgG3 mAb negative control from AbD serotec (Oxford, UK) was used as a negative control (2). Representative positive staining of mAb 8B6 on melanoma (1) representative negative staining of control IgG3 (2) on melanoma cells (G). Photos are reproduced with the permission of Lifespan Biosciences. Magnification 400×. (TIF) [file pone.0025220.s002.tif]

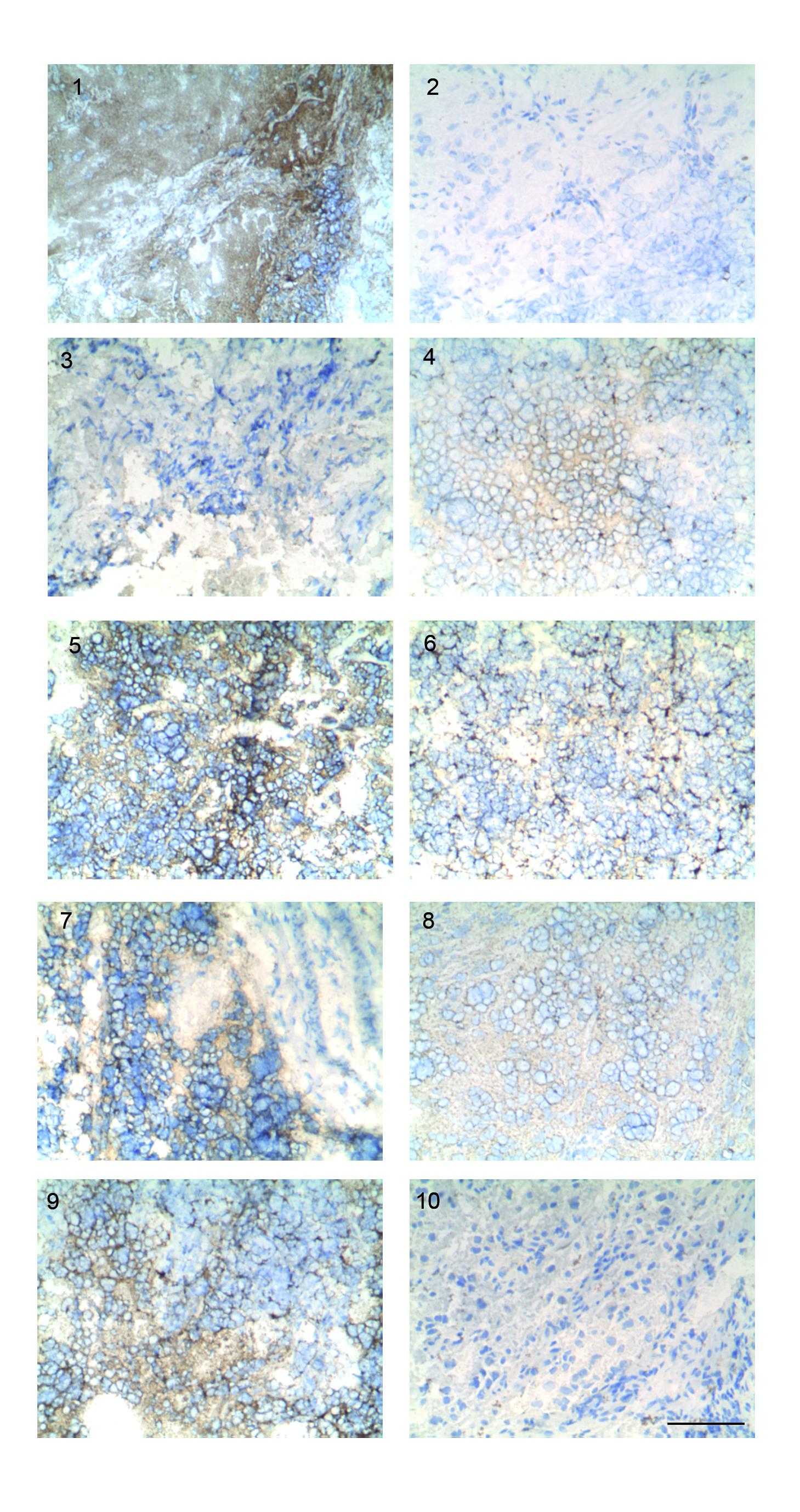

Supplement: Figure S3 — While Table 2 summarize the expression of O AcGD2 on human neuroblastoma tumors, Fig. S4 shows 8 representative examples of O AcGD2 expression on neuroblastomas detected by an immunoperoxydase assay performed with mAb 8B6 as described in the Material and Methods Section. 1, representative positive staining obtained with anti-GD2 mAb; 2, representative negative staining obtained with the mouse IgG3 mAb negative control (AbD Serotec); 3 to 10, representative diversity patterns from neuroblastoma samples. Antibody 8B6 showed moderate to strong positive staining with all neuroblastomas. The percentage of tumor cells that were positive was 100% and staining was both membranous and cytoplasmic. When the corresponding normal cell types were present in the same cancer samples, they were negative. All samples were also positively stained with mAb 14G2a and negative with irrelevant control mAb (data not shown). Scale bar = 100 µm. (TIF) [file pone.0025220.s003.tif]

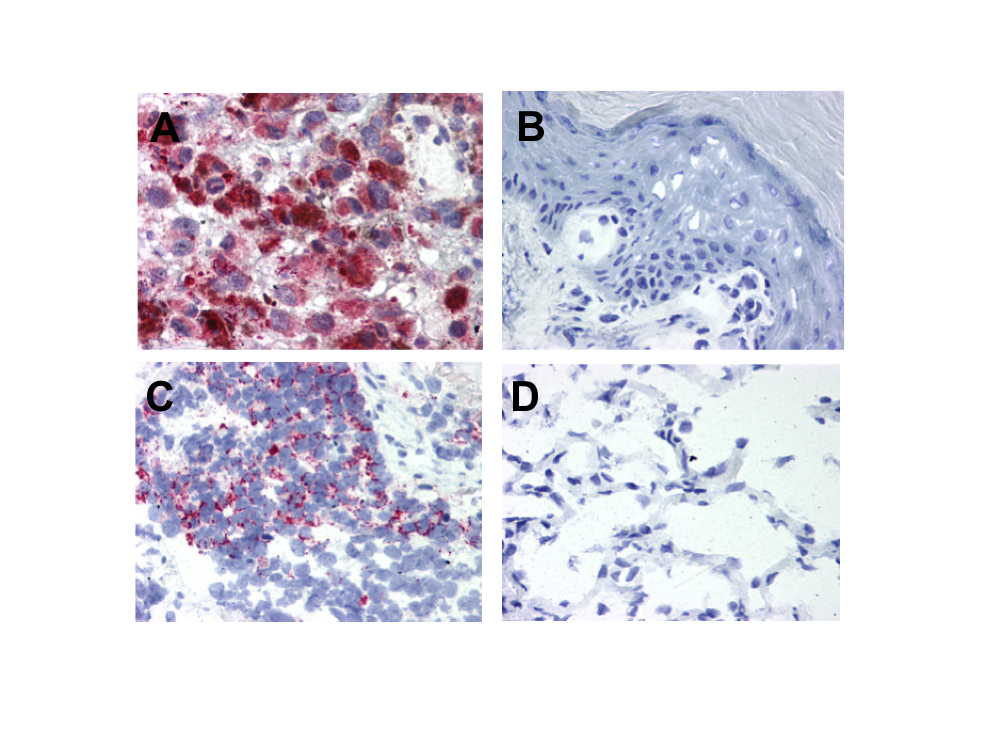

Supplement: Figure S4 — While Table 2 summarize the expression of O AcGD2 on human tumor, in Fig. S4 representative example of expression of mAb 8B6 epitope in melanoma cells (A) and small cell lung carcinoma cells (C) are shown. Skin (B) and lung (D) samples from control patients without cancer showed negative staining in the precursor cell type. The samples were stained with mAb 8B6 using an immunoperoxydase assay performed as described in the “Material and Methods” section. The mouse IgG3 mAb negative control from AbD serotec (Oxford, UK) was used as a negative control (data not shown). Photos are reproduced with the permission of Lifespan Biosciences. Magnification 400×. (TIF) [file pone.0025220.s004.tif]

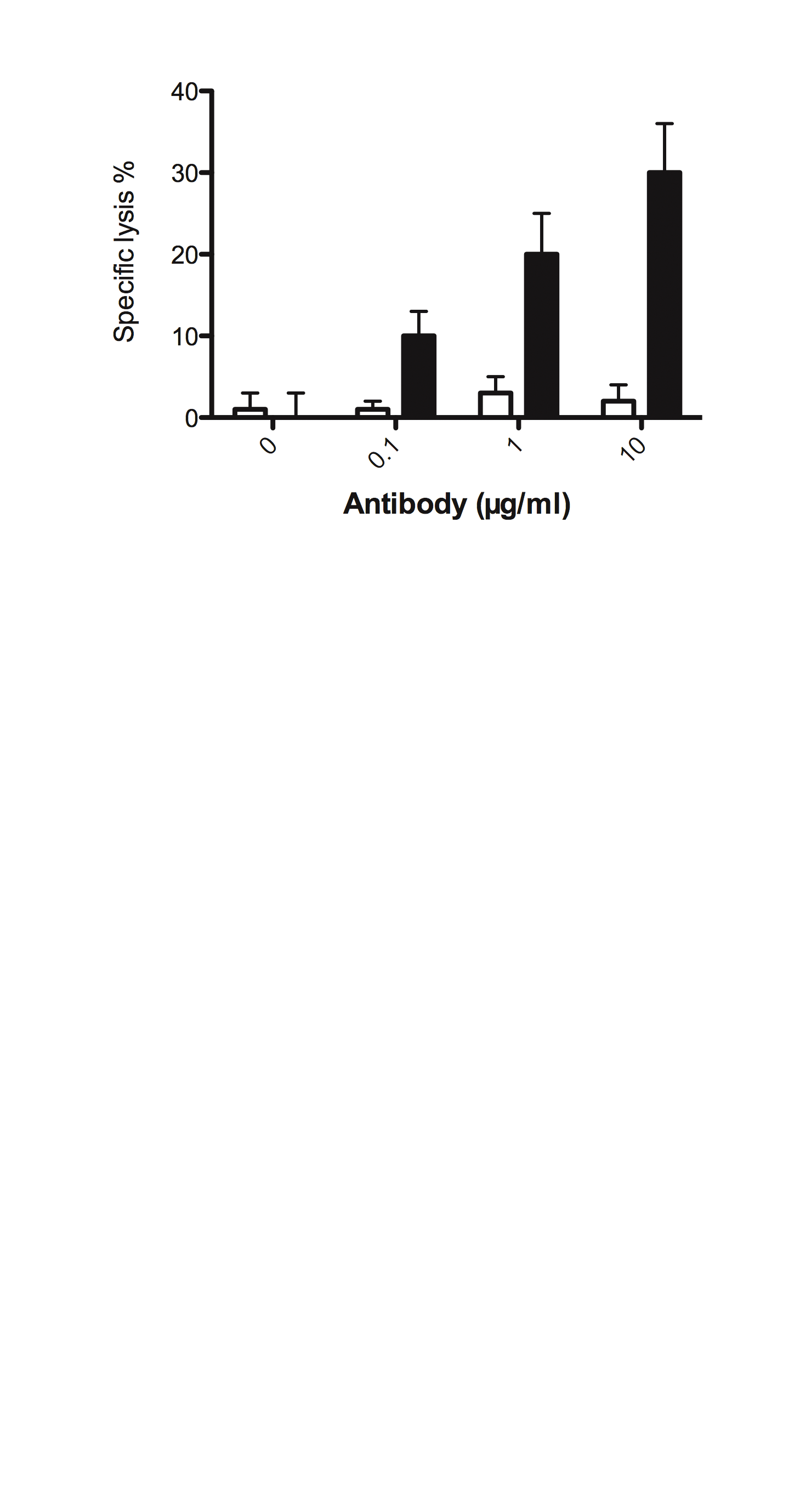

Supplement: Figure S5 — While Fig. 4 shows that mAb 8B6 ADCC activity against mouse EL4 target cells was poorly effective with mouse effector cells, an efficient killer activity of human NK cells with mAb 8B6 is depicted in Fig. S5. ADCC assays were performed at the E/T ratio 50 to 1 using mAb 8B6 (10 µg/ml, black column). Antibody 8B6 specific antibody-dependent lysis occurred in a dose-dependent fashion. Specificity was demonstrated by comparing the ADCC results of mAb 8B6 with non-specific controls using anti-GD3 mAb (10 µg/ml, empty column), which did not show any significant activity, n = 3. (TIF) [file pone.0025220.s005.tif]

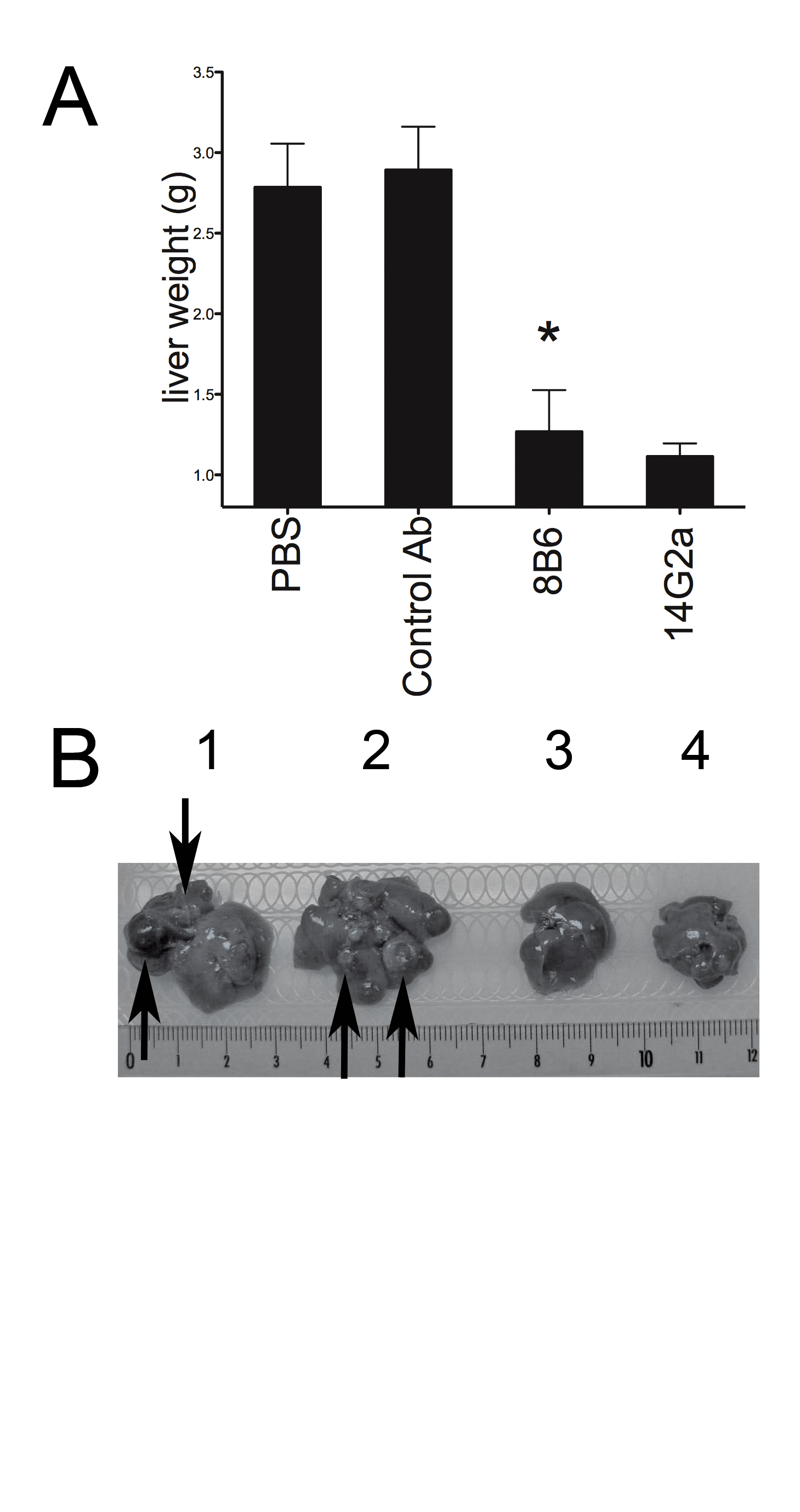

Supplement: Figure S6 — While Fig. 5 shows the anti-tumor efficacy of mAb 8B6 against EL4 lymphoma cells in C57BL/6 mice, the anti-neuroblastoma activity of mAb 8B6 against established experimental liver metastasis are depicted in Fig. S5. Mice (n = 9) were inoculated with 0.25×10^6^ NXS2 cells by i.v. injection and then treated 3 days latter with 5 daily i.v. injections of either 100 µg mAb 8B6, 14G2a and irrelevant antibody. Mice were sacrificed 28 days after tumor cell inoculation. (A) The liver weight was determined on fresh specimen. The y-axis starts at 0.8 g corresponding to the average normal liver weight. The differences in average liver weights between experimental groups treated with mAb 8B6 and mAb 14G2a and all control groups (PBS, control antibody) was statistically significant (* p<0.001). (B) Representative liver specimen of each experimental group (n = 9) are shown. 1, PBS; 2, control IgG3, 3, mAb 8B6; 4, mAb 14G2a. Arrows indicate the location of macroscopic liver metastases. (TIF) [file pone.0025220.s006.tif]
